# How to Predict the p*K*_a_ of Any Compound in Any Solvent

**DOI:** 10.1021/acsomega.2c01393

**Published:** 2022-05-09

**Authors:** Michael Busch, Ernst Ahlberg, Elisabet Ahlberg, Kari Laasonen

**Affiliations:** † Department of Chemistry and Material Science, School of Chemical Engineering, Aalto University, Kemistintie 1, 02150 Espoo, Finland; ⊥ Universal Prediction AB, 42677 Gothenburg, Sweden; § Department of Pharmaceutical Biosciences, Uppsala University, Husargatan 3, 75124 Uppsala, Sweden; ∥ Department of Chemistry and Molecular Biology, University of Gothenburg, Kemigården 4, 41296 Gothenburg, Sweden

## Abstract

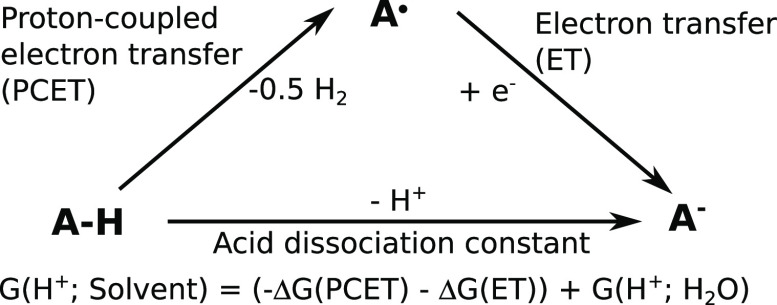

Acid–base properties of molecules in nonaqueous solvents are of critical importance for almost all areas of chemistry. Despite this very high relevance, our knowledge is still mostly limited to the p*K*_a_ of rather few compounds in the most common solvents, and a simple yet truly general computational procedure to predict p*K*_a_’s of any compound in any solvent is still missing. In this contribution, we describe such a procedure. Our method requires only the experimental p*K*_a_ of a reference compound in water and a few standard quantum-chemical calculations. This method is tested through computing the proton solvation energy in 39 solvents and by comparing the p*K*_a_ of 142 simple compounds in 12 solvents. Our computations indicate that the method to compute the proton solvation energy is robust with respect to the detailed computational setup and the construction of the solvation model. The unscaled p*K*_a_’s computed using an implicit solvation model on the other hand differ significantly from the experimental data. These differences are partly associated with the poor quality of the experimental data and the well-known shortcomings of implicit solvation models. General linear scaling relationships to correct this error are suggested for protic and aprotic media. Using these relationships, the deviations between experiment and computations drop to a level comparable to that observed in water, which highlights the efficiency of our method.

## 1 Introduction

Acid–base chemistry is by far the most common class of chemical reactions and of critical importance to almost all areas of chemistry. Examples range from common reactions like the acid- or base-catalyzed hydrolysis of esters to complex processes such as the industrial electrosynthesis of chlorate^1−3^ and renewable energy carriers,^4−11^ the chemistry of nanoparticles,^11−15^ and the transport of pharmacologically active compounds through biological barriers.^16−18^ Indeed, virtually all chemical processes, even those taking place in environments which are generally not associated with acid–base reactions, like aprotic solvents^19−23^ or even the gas phase,^24^ are often affected by the Brønstedt acidity of some of the compounds involved in the process.

Despite the ubiquity of acid–base chemistry, our knowledge of acid–base reactions in solvents other than water is still restricted to the p*K*_a_ of a rather limited number of compounds in the most common solvents.^20,25−29^ But even this fundamental property is not necessarily known accurately. For picric acid for example, p*K*_a_ values of 13.7^29^ and 45^30^ have been suggested in 1,2-dichloroethane. Furthermore, it was suggested that reactions between acids and bases, at least in apolar or aprotic solvents, should not proceed via free solvated protons but instead require direct interactions between the functional groups.^30^ Indeed, developing a fundamental understanding of acid–base chemistry in nonaqueous solvents and predicting scaling relations between the p*K*_a_ values of the same compound in different solvents are still the topic of intensive research.^19,20,26,30^ Considering the difficulties associated with measuring or computing p*K*_a_ values in nonaqueous solvents, these limitations are not surprising. Experiments for example are affected by the almost unavoidable presence of traces of water in the solvent,^20,31,32^ the formation of ion pairs,^20^ and the need to convert measured relative p*K*_a_ values to a common absolute scale.^20^ This manifests itself for example in the unphysical prediction that protons in nonaqueous solvents such as dimethyl sulfoxide (DMSO) should be more stable than their counterparts in water^33−35^ or in significant deviations in the p*K*_a_ values obtained in different experiments.^29^

These experimental limitations in turn have a strong impact on the accuracy of p*K*_a_ predictions from computations. Independent of the exact procedure, state of the art computational methods always rely on experimentally measured p*K*_a_ values in the solvent of interest. Taking for example neural network or machine learning based approaches, which allow for the efficient prediction of p*K*_a_ values at minimal cost, a large data set of reliable experimental values is required to train the algorithm.^25,36,37^ Quantum chemistry based p*K*_a_ prediction protocols such as the isodesmic method, which is also referred to as ”indirect method”,^38−41^ or the direct method,^42−45^ in principle require significantly fewer experimental data points. For example, within the direct method, the p*K*_a_ is predicted using elaborate Born–Haber cycles where the acid dissociation is divided into three steps. Step 1 comprises the transfer of the reactants from the solution phase into the gas phase, which is followed by the chemical reaction (step 2) and the transfer of the products back into the solution phase (step 3).^42^ The Gibbs free energy of the proton in the gas phase formed during step 2 is estimated from the Sackur–Tetrode equation, while the exact proton solvation energy is used for step 3. This value can be computed from the experimental p*K*_a_ but is, owing to the high computational costs and the limited amount of reliable experimental data, only known for very few common solvents.^33,46^ Naturally, such a scheme also requires accurate solvation energies for the acid and its corresponding base which are computed using for example an explicit cluster solvation model^42−44^ or ideally molecular dynamics simulations.^47^ Constructing these models is a routine task in water but becomes, owing to the larger size of the solvent molecules and less obvious interaction geometry, a significant challenge in most nonaqueous media.

These complications can be avoided with the isodesmic procedure, which is probably the most common quantum chemistry based method for p*K*_a_ prediction in nonaqueous solvents.^28,45,48−50^ Within this procedure, a connection to the absolute p*K*_a_ scale is made through experimental reference systems in the solvent of interest. To avoid the need for a demanding explicit solvation model, either a large number of reference systems, which are chemically similar to the compounds of interest,^38,38,51^ or empirical scaling relations^52−55^ are used to correct shortcomings of the implicit solvation models.

An interesting alternative which does not require an experimental p*K*_a_ in the solvent of interest are metadynamics simulations.^56,57^ Owing to the computational costs and complexity associated with the required molecular dynamics simulations this method is, however, not suited for large-scale screenings. Furthermore, the proton solvation energy can in principle also be predicted in any solvent by using the proton in a vacuum as a reference state and computing the energy of the relevant solvent–proton complex.^58−64^ The obtained proton solvation energy can then be used to predict p*K*_a_ values through a direct scheme. Unfortunately, constructing suitable models for the solvated proton is far from trivial, which limits the usefulness of this approach. Similarly, also the ”electrostatic transform method”^26,65^ which connects the p*K*_a_ in two solvents through a gas phase reference could be used if no experimental p*K*_a_ values are available. However, this method requires the knowledge of the proton solvation energy in both media, which is again only known in a rather limited number of common solvents.

A simple yet truly general procedure to predict the p*K*_a_ in a freely chosen solvent that only requires a measured p*K*_a_ in water but does not require experimental data in the solvent of interest or a-priori knowledge regarding the detailed structure of the solvent-proton complex or its solvation energy is, to the best of our knowledge, still missing.

In what follows, we will describe a general computational protocol which allows us to predict acid dissociation constants of any compound in any solvent using only a few standard quantum-chemical calculations and an experimental p*K*_a_ measured in water. In contrast to other approaches, no knowledge of experimental p*K*_a_ values in the solvent of interest or complex computations to construct proton–solvent complexes or molecular dynamics simulations are needed. This method is benchmarked by comparing the predicted p*K*_a_ with experimental values in selected nonaqueous solvents and water. While we find good agreement between predicted and experimental values for all protic solvents, partly significant deviations are observed for aprotic media. These deviations were the result of both experimental uncertainties and the need to use modified scaling relations to correct for the shortcomings of the implicit solvation model used in the present study. This shortcoming can, however, be avoided by the use of more advanced solvation models.

## 2 Methods

### 2.1 Computational Details

All density functional theory (DFT), ab initio (MP2), and semiempirical (PM7^66^) computations were performed using Gaussian 16 Rev B.01.^67^ For DFT calculations, a triple-ζ 6-311++G** basis set with diffuse and polarization functions on all atoms in combination with an ultrafine grid was employed, while the Def2-TZVPP basis set was used for MP2 computations. The influence of the DFT functional was tested by comparing results obtained with B97-D,^68^ PBE0,^69,70^ and M06-2X.^71^ For PBE0, dispersion interactions were included using Grimme’s D3 corrections.^72^ Solvation effects were modeled with the SMD solvation model.^73^ Additionally, the PCM^74,75^ solvation model also was tested in combination with the PBE0 functional. Geometries were optimized for all DFT methods, MP2 and PM7. Entropy and zero-point energy contributions were extracted from frequency calculations at the converged structures. In light of the very large number of computations performed in this study, structures were assumed to be converged when the lowest imaginary frequency was larger than −100 cm^–1^.

Despite the use of a wide selection of DFT and ab initio methods in practice, only a simple functional combined with a sufficiently accurate implicit solvation model is needed for the p*K*_a_ prediction in all solvents and to compute the effective proton solvation energies. Note that the most accurate guess for the proton solvation energy obtained with CCSD(T)/aug-cc-pvqz should not be used for p*K*_a_ prediction if this setup is not explicitly used for the computation of p*K*_a_ values. Instead, the effective proton solvation energy obtained with the chosen methodology should be used as this allows for optimal cancellation of errors. On the basis of this initial benchmark, M06-2X in combination with SMD was selected for the computation of the p*K*_a_ in nonaqueous media since it offered the best combination of accuracy, reliability, and cost. The temperature dependence of the computed p*K*_a_ values was neglected since all comparisons in the benchmark study were made using experimental and computed data obtained under standard conditions.

Following earlier work, the dissociation of formic acid to formate and a proton was used as a reference reaction.^34,76^ This reaction has a p*K*_a_ of 3.77.^77^ Experimental data for the benchmark were taken from the literature.^19,29,51,77−104^

The statistical analysis of the data was performed using the python3 statsmodels package (version 0.12.2–1). All fits were made using the least absolute deviation (LAD) method to reduce the influence of outliers. Cross validations were performed by randomly splitting the data set into five subsets. Consistency was tested by performing three cross validation runs per data set. In what follows, only the results of the run with the highest standard deviation is discussed. All other results can be found in the Supporting Information (SI). Cross validation studies were performed only if the data set had at least 14 points (*n* ≥ 14). Even for the smaller data sets, the different runs are still in reasonably good qualitative agreement. Outliers in the experimental data were not removed, but their effect on the overall fits is limited by the use of the LAD method for fitting. In case several experimental data points were available, the arithmetic mean of all measurements was used; i.e., no assumption regarding their quality was made.

### 2.2 Benchmarking the Computational Setup

Equivalent to previous work, we employed the isodesmic method to predict p*K*_a_ values in water. This method typically allows for the prediction of p*K*_a_ values with chemical accuracy^38,39^ and can even be used in combination with semiempirical methods.^51^ Unfortunately, the success of this procedure depends significantly on the chemical similarity between the reference and the compound of interest.^38,39,51^ It is therefore not uncommon to use a large number of carefully selected reference molecules. While this renders the isodesmic procedure highly accurate, it is at the same time inefficient when dealing with large data sets. A possibility to avoid this limitation is the use of empirical linear free energy scaling relations (LFESR) to correct for the shortcomings of the solvation model.^52−55^ These relationships typically depend on the computational setup and functional group.^40^ To develop a suitable set of LFESRs, we constructed a training set consisting of 113 compounds (159 p*K*_a_ values) which contains both simple and complex organic acids and bases (primary/secondary/tertiary amines, imines, carboxylic acids, alcohols and thiols) whose p*K*_a_ values have been determined experimentally. Additionally, also simple mineral acids, water, and ammonia were considered. For complex molecules with multiple protonation sites, all reasonable protonation states and configurations were tested at the PBE0/SMD level of theory. Only the most stable configurations were considered in the subsequent computations using other computational setups. A summary of all computed p*K*_a_ values and the most stable configurations can be found in the Supporting Information.

**Figure 1:**
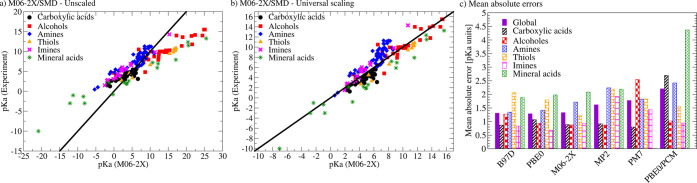
Performance of the isodesmic method for computing p*K*_a_ values for different DFT functionals, the semiempirical PM7 method, and MP2 measured by mean absolute errors (MAE). A summary of the standard deviations can be found in the Supporting Information. (a) Comparison of computed and predicted p*K*_a_ values using M06-2X/SMD without universal scaling corrections; (b) comparison of computed and predicted p*K*_a_ values using M06-2X/SMD with universal scaling corrections (eq 1); (c) summary of mean absolute errors for different computational setups after applying universal scaling corrections (eq 1).

Using only the isodesmic method in combination with M06-2X/SMD, we find that the p*K*_a_ of compounds with similar acid strengths are predicted with fair accuracy, while large deviations are observed for all other compounds (Figure 1a). For stronger acids, the predicted p*K*_a_ is typically too low, whereas it is too high for stronger bases. In contrast to what has been proposed earlier,^40^ we find that these trends are to a large extent independent from the exact nature of the functional group. Considering for example water, HVO_4_^2–^, and ethanol, which all have similar experimental p*K*_a_ values, our computations consistently predict p*K*_a_ values of roughly 25. These shortcomings are a result of the implicit solvation model and can be corrected by applying a linear correction.^52−55^ Using computational method dependent but otherwise universal linear corrections, we indeed find significant improvements for all computational setups. Note that these scaling relations are rather similar for all MP2, PM7, and the DFT methods provided that the same solvation model is used. Significant variations are on the other hand observed between PCM and SMD. Comparable scaling relations between different DFT functionals were also reported in earlier studies.^28^ In the case of M06-2X/SMD for example, the global error measured by the mean absolute error (MAE) decreases from 3.2 p*K*_a_ units (standard deviation (σ) = 4.2 p*K*_a_ units) for the uncorrected p*K*_a_ values to only 1.3 p*K*_a_ units (σ = 1.7 p*K*_a_ units; Figure 1c), which is in the range of acceptable errors for the prediction of p*K*_a_ values.^39^ A similarly low error with a mean absolute error of 1.2 p*K*_a_ units (σ = 1.5 p*K*_a_ units) is also observed for a test set of 20 compounds (29 p*K*_a_ values) which was not included in the original training set. Analyzing the data in more detail we find that the p*K*_a_ values of amines and imines are typically slightly overestimated, while they are underestimated for mineral acids and thiols (Figure 1b). This has, however, only a minor influence on the MAEs associated with the different functional groups, e.g., the mean absolute error increases to roughly 2 p*K*_a_ units for primary and secondary amines and oxo anions but at the same time also decreases to 1 p*K*_a_ unit or less for carboxylic acids, alcohols, tertiary amines, and imines (Figure 1c). Having said this, the error decreases further by using functional group dependent scaling relations in average 0.7 p*K*_a_ units (σ = 1.1 p*K*_a_ units). Similarly, low MAEs are also observed for B97-D and PBE0. MP2 and PM7 display slightly larger global MAEs when being used in combination with method dependent but otherwise universal LFESRs; e.g., the mean absolute error increases to 1.6 p*K*_a_ units (σ = 2.0 p*K*_a_ units) for MP2 and 1.8 p*K*_a_ units (σ = 2.5 p*K*_a_ units) for PM7. Using, however, functional group dependent scaling relations, the mean absolute error of MP2 and PM7 again decreases to a level comparable to that observed for M06-2X/SMD. The worst performance is finally obtained for PBE0/PCM (MAE = 2.2 p*K*_a_ units; σ = 2.8 p*K*_a_ units).

Weighing the cost of the different computational setups, their accuracy, and reliability, we opted for using the M06-2X/SMD setup which reliably predicts the p*K*_a_ for all considered compounds with high accuracy. Initially, shortcomings of the SMD solvation model were corrected using a universal scaling approach (eq 1) to map the computed p*K*_a_ (p*K*_a_(Calc)) to the experimental p*K*_a_ (p*K*_a_(Exp)). This method was used owing to its convenience and the fact that it is still unknown to which extent the scaling relations obtained for different functional groups in water also hold in nonaqueous solvents.
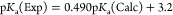
1Evaluation of the validity of this scaling relation in solvents other than water is part of this work.

## 3 Results and Discussion

### 3.1 p*K*_a_ Prediction in Nonaqueous Solvents

p*K*_a_ values in nonaqueous solvents are typically predicted from first-principles calculations using the isodesmic method.^28,45,48−50^ Unfortunately, experimental data for p*K*_a_ values in nonaqueous solvents, which could be used as a reference, are rather scarce in the literature,^20^ and those values which have been reported are sometimes of questionable accuracy (see comparison of experimental data in Supporting Information and the discussion below). Thus, this procedure is restricted to the most common solvents such as methanol, ethanol, dimethyl sulfoxide (DMSO), or acetonitrile.^19,20,29^ A possibility to avoid this limitation is the combination of the isodesmic method^38−41^ with our recently developed procedure for computing absolute potentials of the standard hydrogen electrode (SHE) in nonaqueous solvents.^34^ The isodesmic method relies on the experimentally determined p*K*_a_ of a reference acid (p*K*_a__ref_) to compute the p*K*_a_ through eq 2:^38−41^
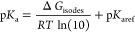
2*R* is the general gas constant, and *T* is the temperature. The Gibbs free energy of the isodesmic reaction (Δ*G*_isodes_) is obtained by assuming a proton transfer from the acid of interest (AH) to the corresponding base of the reference compound (B^–^; reaction 3).
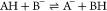
3Here, the p*K*_a_ of the reference acid and its corresponding base serve, identical to the (relative) proton solvation energy (*G*_eff_(H^+^)) typically taken from literature,^33,42,46,105^ the purpose of connecting the relative p*K*_a_ scale to an absolute scale.

Naturally, this connection can only be made if sufficiently reliable p*K*_a_ values are available in the solvent of interest, which effectively limits the procedure to water and the most common nonaqueous media. This problem can be circumvented by combining an effective proton solvation energy (*G*_eff_(H^+^)) with a conversion factor  to adjust it to the solvent of interest, which is here referred to as “SolvX” (eq 4).
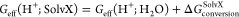
4By using eq 4, the question of predicting p*K*_a_ values in nonaqueous solvents is reduced to two already solved problems: the prediction of the (effective) proton solvation energy in water^76^ and the computation of conversion factors for the solvation energy in all other solvents.^34^

The effective proton solvation energy is easily computed from the p*K*_a_ of the reference compound in water through eq 5:
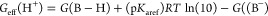
5

Here, *G*(B–H) and *G*(B^–^) correspond to the total energies of the reference acid B–H and its corresponding base. The conversion factor between SolvX and water on the other hand is equivalent to the conversion factors previously introduced for the absolute potential of the standard hydrogen electrode (SHE).^34^ The absolute potential simply corresponds to the energy required to reduce a dissolved proton at pH 0 to gas phase hydrogen using an electron in its resting state in a vacuum.^106,107^ Accordingly, only the proton solvation energy changes when varying the solvent, which is captured by our previously introduced correction factor. Having said this, it is obvious that this value can also be used to convert the effective proton solvation energy in water, computed using eq 4, to that in the solvent of interest. In what follows, we will introduce this conversion factor and quickly reiterate the central steps for deriving the central equations. A more detailed discussion can be found in ref (34).

The first step in obtaining the conversion factors is to rewrite the dissociation of the reference compound into its corresponding base and a dissolved proton by a simple thermodynamic cycle, which consists of a proton-coupled electron transfer (PCET) oxidation (reaction 6)
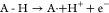
6followed by an electron transfer (ET) reduction (reaction 7).
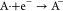
7The electrochemical potential associated with the PCET step can be computed using the “computational standard hydrogen electrode”^108^ which uses the formation of H_2_ from H^+^ and an electron as reference reaction (reaction 8) to connect it to the SHE scale.
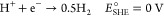
8Accordingly, the electrochemical potential of the PCET reaction is given by eq 9
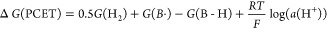
9*G*(H_2_) corresponds to the total energy of hydrogen in the gas phase and *G*(B·) and *G*(B–H) to the total energies of the reactants and products in the solvent of interest. *F* is the Faraday constant. The term (*RT*/*F*) log(*a*(H^+^)) introduces, identical to the Nernst equation, the pH dependence of the PCET step and is 0 under standard conditions, i.e., if the proton activity *a*(H^+^) = 1 mol/L.

Electrochemical potentials of ET reactions on the other hand are typically obtained from the (effective) absolute potential of the SHE (*E*_abs_(SHE))) in water through eq 10.^76,109−111^
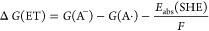
10

Assuming this Born–Haber cycle, the Gibbs free energy of the acid dissociation (Δ*G*(Diss)) in water simply corresponds to the sum of the Gibbs free energies of the PCET (Δ*G*(PCET)) and ET (Δ*G*(ET)) steps (eq 11).
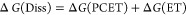
11Let us now assume we are interested in predicting the p*K*_a_ in an arbitrary solvent X, such as cyclohexane, while the p*K*_a_ of our reference system is only known in water. In this case, we are neither able to compute the p*K*_a_ through the isodesmic method nor the direct method since we lack a reference system in cyclohexane and do not know the energy of solvation of the proton in this solvent. A possibility to circumvent this limitation is the use of the above-discussed thermodynamic cycle which splits the acid dissociation into a PCET and an ET step. In nonaqueous solvents like cyclohexane, eq 11 is no longer directly applicable since the computational standard hydrogen electrode predicts the potential of the PCET step versus the SHE in cyclohexane, while the ET step is, owing to the use of the absolute potential in water, predicted versus the SHE in water. Note that from a purely formal perspective both SHE references can be used since the IUPAC definition of the SHE does not specify a solvent.^112^ Owing to these different SHE references, the hypothetical dissociation of the reference acid no longer corresponds to the sum of PCET and ET steps and needs to be rewritten according to eq 12.
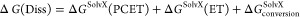
12This correction corresponds to the conversion factor between the SHE in different solvents introduced by us previously.^76^

The conversion factor can be obtained from assuming a proton dissolved under standard conditions in the solvent of interest. Since the proton has a p*K*_a_ of 0, it is in equilibrium under these conditions. This is again valid in all solvents since the definition of the p*K*_a_ again does not refer to any specific solvent.^112^ Accordingly, the dissociation energy in eq 12 becomes
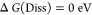
13and the conversion factor can be computed by rearranging eq 12 to eq 14:
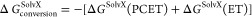
14Inserting the equations for the PCET and ET steps (eqs 9 and 10), the conversion factor is finally given by eq 15:
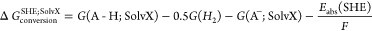
15

Having computed the absolute potential of the SHE in the solvent of interest, the effective Gibbs Free energy of the proton is easily predicted by assuming reaction 8 under the assumption that the hydrogen evolution reaction has a potential of 0 V under standard conditions, i.e., the effective total energy of the proton in a freely chosen solvent  is given by eq 16.
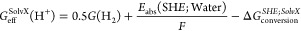
16

### 3.2 H^+^ Solvation Energy in Nonaqueous Solvents

**Figure 2:**
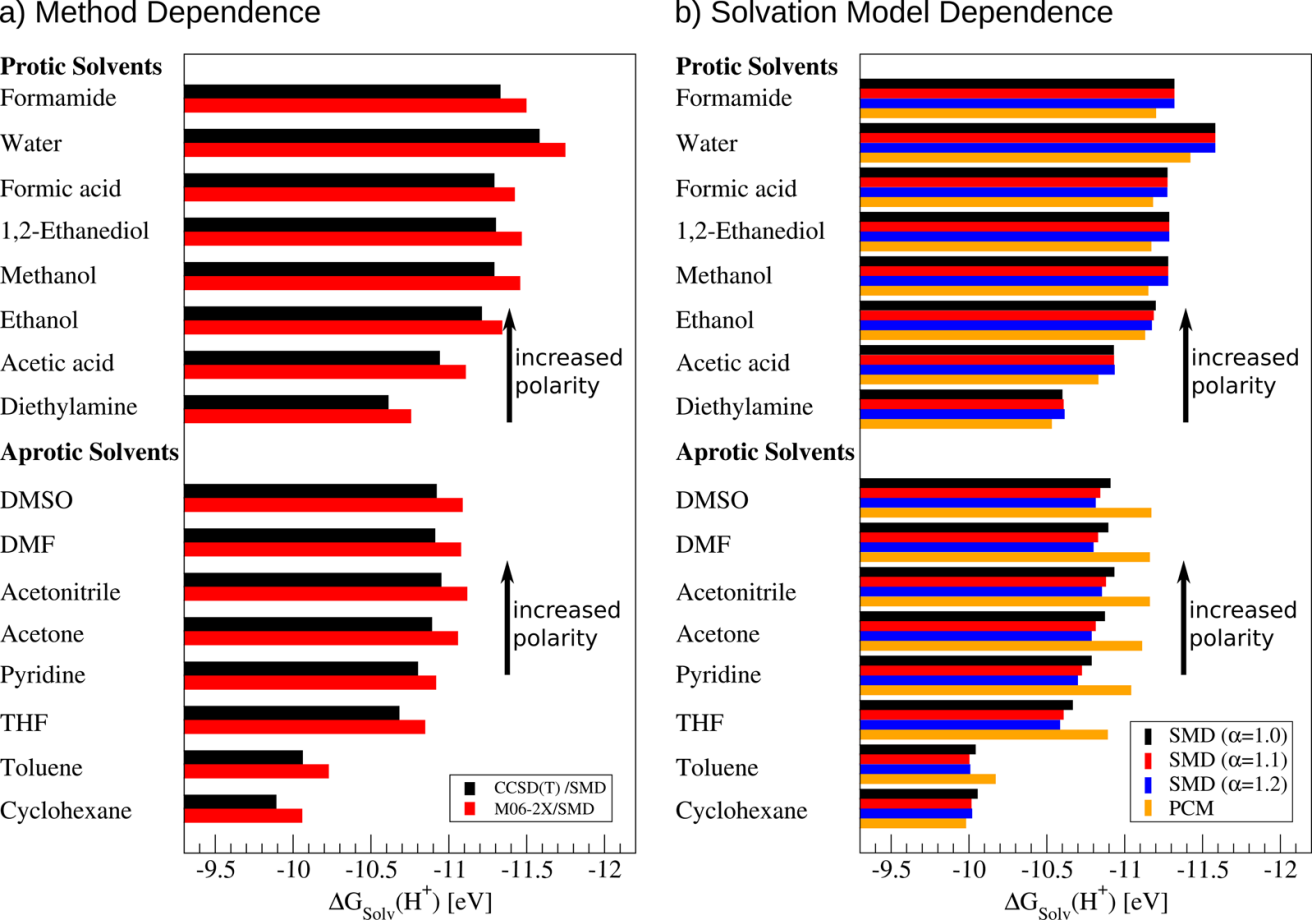
Proton solvation energy in different protic and aprotic solvents. The values were in part computed based on the raw data presented in ref (34). All computations were performed using CCSD(T)/Def2-TZVPP if not stated otherwise and formic acid was used as a reference reaction in all cases. The values were obtained using eq 16. (a) Dependence on the quantum-chemical method; (b) influence of the implicit solvation model.

Taking advantage of our new procedure, it is possible to estimate the proton solvation energy in any solvent at a minimal computational cost. A summary of proton solvation energies in selected protic and aprotic solvents can be found in Figure 2. The total energies of the proton in different solvents were computed using eq 16, and the most accurate guesses for absolute potentials of the SHE obtained at the CCSD(T)/Def2-TZVPP/SMD level presented by us earlier.^34^ The obtained total energy of H^+^ is converted to the commonly reported proton solvation energy by subtracting the total energy of H^+^ in a vacuum ((H^+^) = −0.27 eV) estimated from the Sackur–Tetrode equation.^42^

Let us start the discussion with the solvation of a proton in water. Here, our CCSD(T)/SMD computations suggest a solvation energy of  = −11.58 eV, which is in good agreement with the commonly accepted proton solvation energy of −11.53 eV.^42,46,105,113,114^ It must, however, be cautioned that the proton solvation energy is extremely sensitive to the computational setup. For example, even minor changes such as the choice of basis set or the electronic structure method have a significant influence and can easily result in shifts on the order of several 100 mV in the absolute potential,^76^ which in turn translates into comparable changes in the proton solvation energy (see Supporting Information). Furthermore, Δ*G*_solv_(H^+^) can vary between different reference acids owing to the shortcomings in the description of the acid or its corresponding base by the implicit solvation model.

Having said this, it is clear that the success of computing the absolute p*K*_a_ by using the literature value for the proton solvation in a direct p*K*_a_ scheme will also strongly depend on the choice of the computational method. These uncertainties were indeed found in earlier studies.^42,115^ Unfortunately, the absolute potential of the SHE and thus also the proton solvation energy varies randomly upon changing the basis set or electronic structure method, and no clear trends exist which could be exploited to systematically improve the accuracy of the direct p*K*_a_ scheme.^76^

Interestingly, this method dependence cancels for the conversion factors between different solvents. These are robust with respect to the quantum-chemical method and the detailed parametrization of the implicit solvation model (Figure 2). Accordingly, variations between the predicted conversion factors are typically far below 100 meV.^34^ Note that the variations between M06-2X/SMD and CCSD(T)/SMD are mainly a result of the differences in the effective absolute potential in water; e.g., the difference in the solvation energies between the two methods remains approximately identical for all solvents. The stability of the method is the result of the method inherent cancellation of errors in eq 15 which renders the relative differences between solvents robust with respect to the electronic structure method and the choice of parameters for the implicit solvation model. Owing to this error cancellation, also the choice of the acid–base reference couple in water will most likely not affect the conversion factors. Switching on the other hand to a different implicit solvation model such as PCM can significantly affect the computed values (Figure 2b).^34^ These variations are not necessarily a result of the worse performance of the PCM model but rather reflect the fact that SMD predicts, at least in water, the so-called inner or Galvani potential, whereas PCM predicts the experimentally accessible outer or Volta potential.^34,76,111,116^ These two potentials differ by the surface potential χ which is a result of electrostatic interactions at the liquid–gas or liquid–solid interphase which need to be overcome during electrochemical reactions or dissolution.^117,118^ Unfortunately, for most solvents, the magnitude of the surface potential is poorly known.^34,119−123^ This will, however, not affect the prediction of p*K*_a_ values since the surface potential only influences the solvation energies of charged species but cancels for overall charge neutral reactions if included or excluded consistently.^41^

Upon moving from water to other protic solvents such as methanol or formamide, we observe a decrease in the relative stability of the dissolved proton. This is not unexpected when considering that water has a unique ability for hydrogen bonding^124^ and is at the same time highly polarizable, which makes it best suited for stabilizing protons. Other nonaqueous solvents such as formamide or acetic acid may be more polarizable or allow for stronger hydrogen bonding,^125^ but none of them possess the unique combination observed for water. A possibility to describe the complex interplay of polarizability and hydrogen bonding in a simple manner offers linear solvation energy relations such as the Kamlet–Taft equation^126^ Within the Kamlet–Taft model, the change of an arbitrary property is described by the solvatochromic parameters π*, α, and β. α and β correspond to the ability for hydrogen bond donation (Lewis acidity) and acceptance (Lewis basicity), while π* describes the polarizability of the solvent. The molar volume of the solvent is, following earlier work, neglected.^34^ In what follows, the solvatochromic parameters reported by Marcus^125^ are used to establish trends. The solvation energy of protons follows the relationship shown in eq 17 with respect to the solvatochromic parameters.
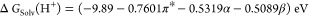
17According to eq 17, protons in nonaqueous solvents are stabilized to a comparable degree by the polarizability of the solvent, hydrogen bond acceptance, and hydrogen bond donation. In line with this, we find that protons are most stable in highly polarizable media, which also possess the ability for strong hydrogen bonding such as formamide, methanol, and water. Moving away from these ideal solvents, solvated protons become significantly less stable. Taking for example acetic acid, which according to the solvatochromic parameters is an excellent hydrogen bond donor but only fairly polarizable, the solvation energy of the proton increases by 0.64 eV to only −10.94 eV (Figure 2). This renders its ability for direct Brønstedt acid–base chemistry through free protons comparable to that of polarizable aprotic solvents such as dimethyl sulfoxide (DMSO), dimethylformamide (DMF), acetone, or acetonitrile. Interestingly, we find that the proton solvation energies in these solvents are, despite their different properties, very similar; i.e., they vary by only 30 meV. This would, in contrast to experimental results^20,103^ indicate that also the p*K*_a_ in these media should be similar. Moving toward even less polarizable protic solvents like dimethylamine, which is only formally a protic solvent but according to the solvatochromic parameters does not possess any ability for hydrogen bond donation,^125^ the proton solvation energy increases even further to only −10.61 eV, which is comparable to rather apolar aprotic solvents such as tetrahydrofuran (THF). Having said this, the use of a nonaqueous protic solvent does not necessarily imply that free protons for Brønstedt acidity are present nor does the use of an aprotic solvent completely exclude it. However, in most practical cases this possibility can, in line with earlier work,^30^ be considered unlikely.

Considering the importance of proton solvation energies, this topic was subject to intensive research, and predictions for several of the here considered solvents can be found in the literature. In what follows, we will restrict the discussion to methanol, acetonitrile, and DMSO for which we were able to identify experimental and computational data from different sources. Let us start the discussion with methanol. Here, DFT modeling suggests that the proton transfer from water to methanol should be approximately thermoneutral,^127^ which in turn implies that also the proton solvation energies should be similar. This is in good agreement with reported proton solvation energies obtained from computational modeling, which vary between −11.31 eV and −11.53 eV^27,33,58−60,128^ and thus are only slightly lower than the proton solvation energy in water. Experiments also indicate similar values, e.g., the proton solvation energy varies between −11.36 eV and −11.54 eV in different studies.^35,106,129,130^ An interesting outlier is finally the proton solvation energy suggested by Pliego et al.^131^ who predicted a solvation energy of only −11.00 eV. This difference is, however, to a significant part the result of the inclusion of the surface potential which is neglected in the other studies cited above. This value can be converted by subtracting the experimentally obtained surface potential energy of roughly 0.2 eV.^119−121,123^ Note that this value assumes the electrochemical definition of the surface potential which has also been suggested by IUPAC.^112^ Here, the outer potential or real solvation energy includes χ and thus must be subtracted. Accordingly the value of Pliego et al. corresponds to a proton solvation energy of approximately −11.2 eV (inner potential or intrinsic solvation energy), which is in fair agreement with other literature values. In our study, we finally predict a proton solvation energy of −11.29 eV for the inner potential or intrinsic solvation energy which is, considering the significant scatter between different studies, in reasonably good agreement with the literature values.

A similarly good agreement is also observed for acetonitrile where our computations indicate a proton solvation energy of −10.95 eV which is at the lower boundary of values obtained from computational modeling. These vary between −10.94 eV and −11.28 eV.^27,33,59,62,63,128^ The much lower values for the proton solvation energies between −10.52 eV and −10.80 eV reported by Malloum et al.^61^ are again a result of the difference in inclusion of the surface potential. Upon correcting these values by the experimental surface potential energy of approximately 0.1 eV,^121,132^ they become reasonably similar to other literature values and our estimate. Note that the spread by approximately 0.3 eV between the different values is a result of the method dependence of the predicted proton solvation energies. Similar effects are likely also responsible for the variations between different studies and were also observed by us (see Supporting Information). In our novel approach, this effect cancels when computing the relative changes through eq 16 and is therefore only present in the proton solvation energy of water which is used as a reference. Experiments finally indicate a similar proton solvation energy between −11.02 eV and −11.07 eV.^35,129^ This significantly lower stability of a proton in acetonitrile compared to that in water also agrees well with the much higher acidity of protonated acetonitrile.^27^

For DMSO, our computations indicate that the proton solvation energy should, in line with chemical intuition, be comparable to that of acetonitrile. Qualitatively equivalent results are observed for all DFT and ab initio methods as well as for the PCM, SMD, and pbf solvation models.^34^ This is, however, in disagreement with earlier work which almost consistently suggests that the proton solvation energy should be similar to that in water or even be somewhat more negative; i.e., modeling results vary between −11.55 eV and −11.85 eV.^27,33,59,62,64,128,133,134^ Comparable results were also obtained experimentally.^35,129^ The only exception is the work of Carvalho et al.^63^ who with a solvation energy of −11.32 eV also predicted that protons in DMSO should be less stable than in water. This is, however, still much higher than the −10.92 eV computed by us. It is noteworthy that these trends are also reproduced by the p*K*_a_ of the protonated DMSO, which has a p*K*_a_ which is similar to that of water^27,135,136^ and independent measurements of the absolute potential.^137^ Experiments comparing the proton transfer kinetics in methanol and DMSO on the other hand indicate that only a direct transfer from the acid to the base without involvement of the solvent is possible.^138^ This speaks in favor of a rather low stability of the DMSO–proton complex as its existence in significant concentration enabled by a more stable proton–DMSO complex would allow for an indirect proton transfer.

In the light of the rather good agreement between our predictions and the literature for methanol and acetonitrile, these qualitative and quantitative differences observed for DMSO are surprising. This is even more so when considering that the only true assumption in our novel procedure is to assume that the chosen computational setup is appropriate, i.e., that CCSD(T) or DFT methods combined with SMD, PCM, or pbf are sufficiently accurate to describe the electronic structure and solvation energy of formic acid and formate. Having said this, the only realistic source of errors could be the failure of the implicit solvation model to correctly describe anions and/or neutral molecules in DMSO. Such a failure can be considered unlikely when taking into account the typically excellent performance of SMD.^139^ Also problems in the parametrization are unlikely since three completely independent methods and also SMD with different cavity sizes make qualitatively the same predictions (see Supporting Information). Similarly, also an error in the reference p*K*_a_ in water can be excluded as this would affect proton solvation energies in DMSO, methanol, and acetonitrile equivalently.

Computations reported in the literature so far on the other hand either rely on a p*K*_a_ value in the solvent of interest^27,33,128^ or a reference state of the proton in the gas phase.^58−64^ The former modeling approach suffers mainly from the uncertainties associated with the determination of the experimental p*K*_a_ values. As shown by us below through statistical analysis, these errors are not so much a result of direct experimental problems like impure solvents but rather due to uncertainties in offsetting the measured relative p*K*_a_ values to a common absolute scale. This problem can be avoided by directly modeling the proton–solute complex in the different solvents and using a proton in a vacuum as reference. However, constructing these complexes without extensive molecular dynamics simulations, as it is commonly done is already in water a significant challenge^47^ and very likely even more so in nonaqueous media. Thus, it is unlikely, that these clusters represent the true complexity of the solvation shell which may result in an increased error bar. Accordingly, differences between the true proton solvation energy and these earlier reported computed values could be expected.

As explained above, our procedure does not suffer from any of the above-mentioned limitations and consistently makes the same predictions independent of the type of solvation model or code it is implemented in (see Supporting Information and Figure 2b). Furthermore, changes to the parametrization of the SMD solvation model have no effect on the global trends between solvents (see Figure 2b). We are therefore rather confident that the trends obtained using our novel procedure indeed correspond to the true trends between proton solvation energies.

### 3.3 Benchmarking p*K*_a_ in Nonaqueous Solvents

Taking advantage of these correction factors for the conversion of proton solvation energies from water to any other solvent, it is possible to compute the p*K*_a_ of any compound in any solvents. The only practical limitation is the existence of a suitable solvation model. The quality of the computed p*K*_a_ is assessed for a test set of 142 molecules by comparing the prediction with experimental data in 12 solvents. The molecules were chosen such that they only possess a single acid–base active site or in case multiple active sites were present, such that all sites are identical due to molecule symmetry. This allows us to avoid problems from possible differences in the deprotonation sequence between the solvents and thus ensures that computed p*K*_a_ values are directly comparable to literature values.

But it must be cautioned that the reported experimental p*K*_a_ values are not necessarily fully reliable. These uncertainties are for example known to result in predictions for the absolute potential of the SHE and the closely related proton solvation energy, which are not chemically viable if the experimental p*K*_a_ in the solvent of interest is used.^34^ Taking for example acetone or DMSO, predictions using the p*K*_a_ in these solvents suggest that protons in these solvents should be equally or more stable than those dissolved in water.^33,34,106^ A similar trend has also been reported for the proton solvation energy in DMSO.^27,33,35,59,62,64,128,129,133,134^ When taking into account that these solvents are, with respect to the combination of polarizability and hydrogen bonding abilities, inferior to water, such a prediction can be considered unlikely and it instead highlights the poor quality of the experimental data on which they are based. It has been suggested that these chemically not viable predictions are due to the presence of traces of impurities such as water which interact either with the acid or its corresponding base.^34^ The influence of water impurities will affect the p*K*_a_ in very apolar aprotic solvents the most since these media are unable to properly stabilize charged species. Similarly, also ion pairing is known to occur in aprotic solvents.^20^ This is neglected in our computations and will result in deviations if not corrected in the experimental p*K*_a_ values. In practice, these factors are, however, only a minor source of errors since they cancel in the relative p*K*_a_ values obtained during the same series of experiments.

**Figure 3:**
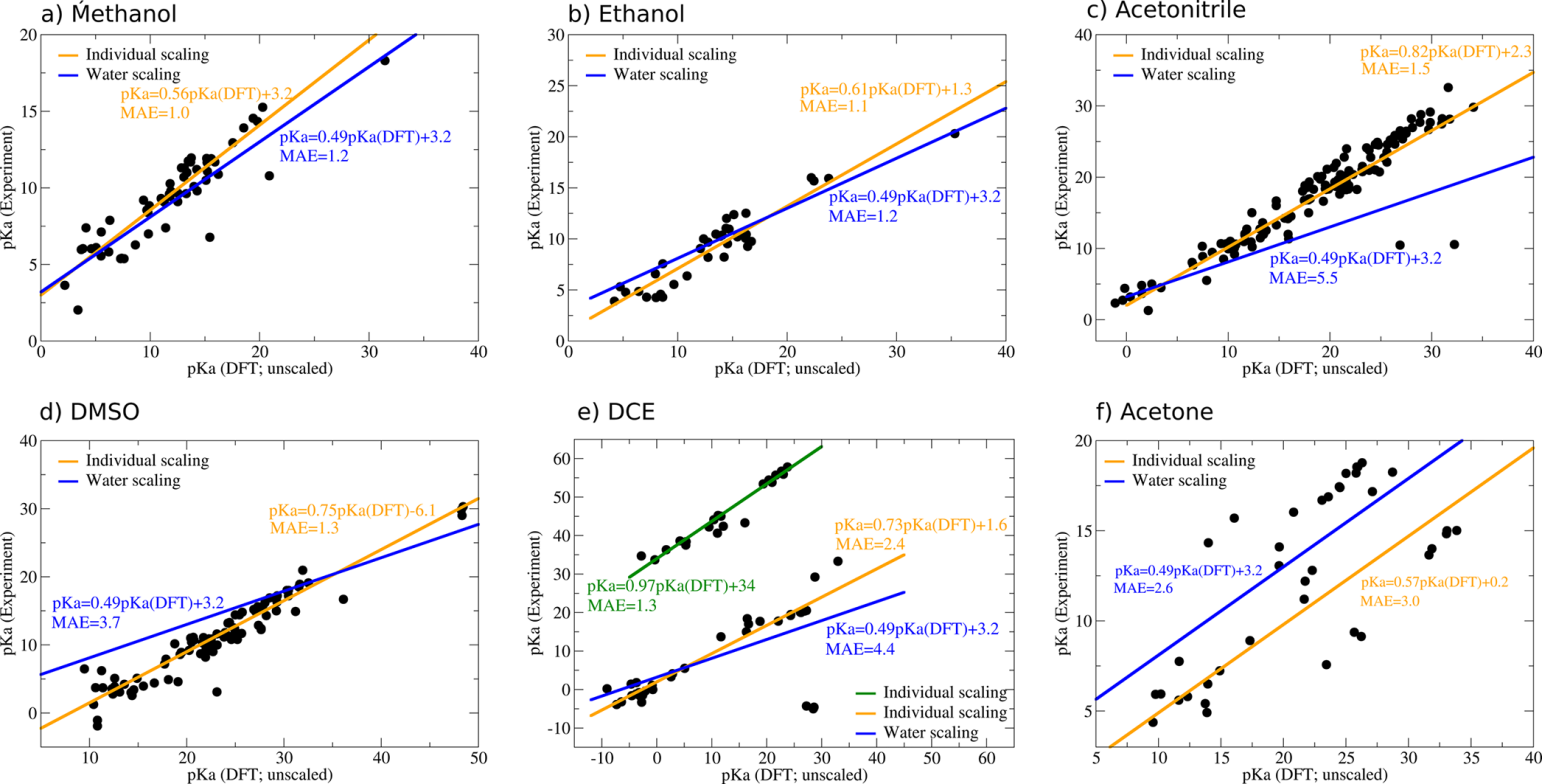
Direct comparison between computed p*K*_a_ values and experiment for selected solvents. (a) Methanol; (b) ethanol; (c) acetonitrile; (d) DMSO; (e) DCD; (f) acetone.

The obtained experimental relative p*K*_a_ values are then converted to an absolute scale using a reference p*K*_a_.^20^ Indeed, this conversion adds significant uncertainty since it requires the knowledge of the exact absolute p*K*_a_ of a reference compound.^20^ Naturally, any errors in the reference p*K*_a_ will introduce a constant error to the absolute p*K*_a_ values. Errors of this type can not be identified easily through comparison of different experiments since it is in this case unclear which of the experiments is correct or whether all are problematic. Additionally, computations using an implicit solvation model can not provide a definite answer since these are known to deviate by a yet unknown linear scaling relation. Instead, the impact of this potential source of errors could only be evaluated by using more complicated explicit models in combination with our above presented method for estimating the proton solvation energy. Owing to the significant computational costs associated with such an ansatz, this is far beyond the scope of this study. Additionally, using inconsistent reference values in different experiments can result in inconsistent experimental p*K*_a_ sets for the solvent of interest. These errors appear as two or more internally consistent but otherwise inconsistent data sets when plotting the unscaled computed and experimental p*K*_a_ values or simply as very different experimental p*K*_a_ values for the same compound in the same solvent. An example of this is picric acid in 1,2-dichloroethane (DCE) which depending on the study either displays a p*K*_a_ of 45^30^ or 13.7.^29^ Similar deviations are also present for other compounds in DCE as indicated by the appearance of at least two internally consistent but otherwise inconsistent data sets (Figure 3e). For less extreme cases, this source of error could also appear as an increase in scatter around the slope in the scaling relation between computations and experiment. This in turn translates into a larger standard deviation, which measures the difference between the predicted and “true” value, and variance, which is a measure of the spread in the data points, at the intercept during the cross validation.

Considering these uncertainties associated with the experimental reference data, it is clear that they can not automatically be considered as ”correct” and thus, directly used. In what follows, we therefore only discuss deviations between experiments and computations as well as qualitative trends in a neutral manner.

**Figure 4:**
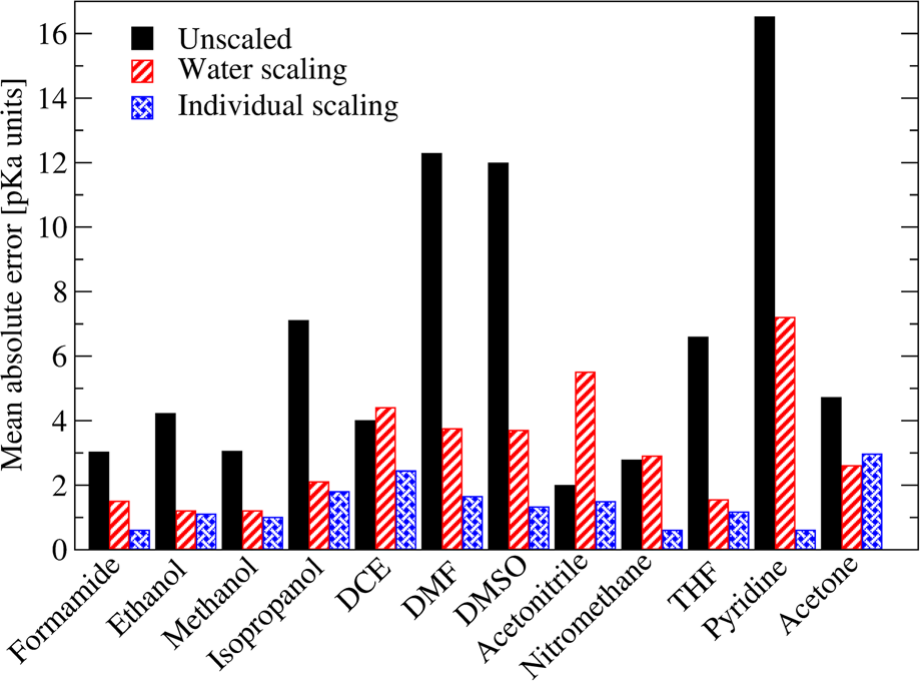
Accuracy of our method for predicting p*K*_a_ values in nonaqueous solvents after scaling with the universal scaling relations obtained for water and individual scaling relations. The experimental reference data was taken from refs (19, 29, 30, and 101−104).

Let us start the discussion with the unscaled computed p*K*_a_ values in selected protic and aprotic solvents (Figure 4). If we consider for example p*K*_a_ values in pyridine and acetonitrile, the average deviation between experiment and computations varies between 16.5 p*K*_a_ units (pyridine) and 2 p*K*_a_ units (acetonitrile). All other solvents are between these two extremes. It is noteworthy that no trends between the solvents and the error associated with the unscaled p*K*_a_ exist. The mean absolute error drops slightly to ∼12 p*K*_a_ units for DMSO and DMF and roughly 7 to 8 p*K*_a_ units for isopropanol and THF. Somewhat lower but still very high values of at least 3 p*K*_a_ units are observed for the remaining solvents. Similarly, high errors have also been observed for unscaled computed p*K*_a_ values in water (Figure 1c) and are a result of shortcomings in the construction of the SMD solvation model.

A possibility to correct for these errors is the use of suitable scaling relationships to map the computed to the experimental p*K*_a_ values. In the light of the problems associated with the experimental data, we initially decided to rely on the global SMD scaling relations obtained for water (eq 1) to scale the p*K*_a_ values in all nonaqueous solvents. Doing so reduces the deviations between experiments and computations in most cases drastically, but they still remain high for most aprotic solvents (∼3–4 p*K*_a_ units; Figure 4). An exceptions to this is acetonitrile where the MAE increases from ∼2 p*K*_a_ units to 5.5 p*K*_a_ units. In DCE (only data points following the orange fit in Figure 3e are considered) and nitromethane, the MAE is approximately identical to the unscaled case. THF finally displays an error which is almost comparable to that observed in water (MAE(THF) = 1.8). The typically poor agreement between experiment and scaled computations for aprotic solvents is contrasted by the protic media. Here, a deviation of only 1.2–1.5 p*K*_a_ units is observed for methanol, ethanol, and formamide (Figure 4). The somewhat larger deviation of 2.1 p*K*_a_ units observed for isopropanol is most likely the result of some inconsistencies in the experimental data; i.e., the comparison between unscaled computed and experimental data hints at the presences of two inconsistent data sets (see Supporting Information).

**Figure 5:**
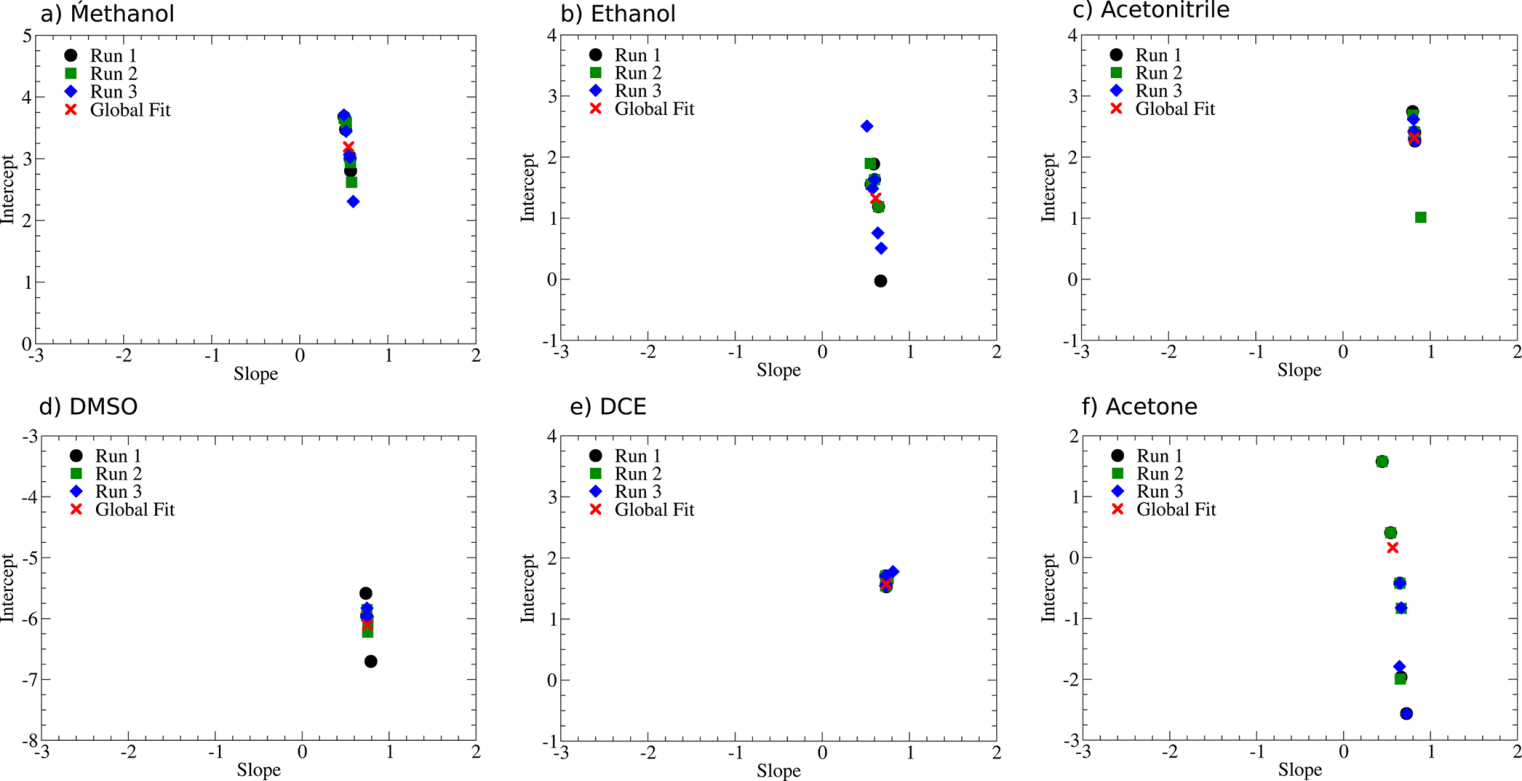
(a–f) Summary of cross validation results. A linear fit was used in all cases, i.e., p*K*_a_(Exp) = Slope*p*K*_a_(DFT) + Intercept. Each cross validation was performed by randomly splitting the data sets into five subsets. A total of three runs were performed. In the text, only the run with the highest standard deviation for slope and intercept is discussed. A complete summary can be found in the Supporting Information.

The significant deviations in the MAEs between aprotic and protic solvents are a result of the very different scaling relations between unscaled computed and experimental p*K*_a_ values (Figure 3). In case of the protic solvents, the correlation is well described by the scaling relation obtained for water (eq 1); e.g., the slope and intercept of the explicit fit for the solvent and those obtained for water are almost identical (Figure 3a and b). Judging from the cross validation study (Figure 5a,b), the fits are reliable as indicated by a very small standard deviation σ of the slope of approximately 0.05 and a variance σ^2^ of ∼0.003. For the intercepts, the deviations between the different fits are with a σ of 0.5–0.8 p*K*_a_ units and a variance between 0.3 and 0.6 units somewhat larger. The very low errors of the slopes indicate that the relative p*K*_a_ values can be obtained to a high degree of accuracy both computationally and experimentally, whereas the somewhat larger deviations in the intercept indicate that the main source of error is the choice of reference to convert the relative p*K*_a_ values to absolute p*K*_a_ values. Note that the uncertainties related to the computational procedure of computing the proton solvation energy will not affect the variance and standard deviation of the intercept as they only enter as a constant shift of the intercept which affects all subsets equally.

Using solvent-dependent scaling relationships for protic solvents typically has only a very minor influence on the mean absolute deviation between experimental and scaled computed data; e.g., it remains approximately the same for ethanol and methanol, while it drops by approximately 0.9 p*K*_a_ units for formamide. The significant difference observed for formamide is not so much a result of the differences in the slope of the water and formamide scaling relation but rather due to the difference of 3 p*K*_a_ units for the intercept. The only exception for the aprotic solvents is finally isopropanol, which, owing to the small number of data points and the potential existence of two inconsistent experimental data sets, displays a significantly lower slope in the solvent-dependent scaling relationship. These uncertainties manifest themselves also in the cross validation through a significantly increased standard deviation (σ(slope) = 0.12; σ(intercept) = 1.54) and variance (σ^2^(slope) = 0.014; σ^2^(intercept) = 2.38). Again, the main source of errors appears to be the uncertainties in the intercept. Overall, the scaling relations for water (eq 1) are sufficiently similar to those of the different protic solvents considered in this study. Thus, the use of the water scaling relations can be justified for protic solvents, where owing to the lack of (reliable) data, no explicit scaling relations can be obtained. Using these scaling relations results in an equivalent error for methanol and only a modest increase for the other protic solvents (see Supporting Information).

Similar to the protic solvents, individual solvent-dependent scaling relations also exist for aprotic media (Figure 3c–f). In contrast to water and the protic solvents we typically find that the slope of the scaling relation between computations and experiments increases to approximately 0.75. The only exception to this is pyridine whose slope is, with a value of 0.46, almost identical to that in water. The rather good agreement for the slope is contrasted by a significant scatter in the intercepts which vary between −6 for DMSO and DMF and 2 for DCE, acetonitrile, and nitromethane. Using these new individual scaling relationships, the mean absolute deviations drop to values between 0.6 and 2.4 with most solvents displaying values of the order of 1.4 p*K*_a_ units (Figure 4).

These scaling relations are, in contrast to the protic solvents, typically much less reliable. The smallest deviations are found in acetonitrile and DMSO, where both the intercept and slope are described well by the fit, e.g., the standard deviations and variances obtained from the cross validation are of the same order of magnitude as those for formamide or methanol. The uncertainties on the intercept significantly increase when moving to nitromethane, where a standard deviation and variance of 1 p*K*_a_ unit is observed for the intercept which increases even further for pyridine and DMF (σ(intercept) ≈ 1.4; σ^2^(intercept) ≈ 2.0). However, even in these cases, the uncertainties in the slope remain still almost negligible (σ(slope) = 0.061; σ^2^(slope) = 0.04). The largest MAEs are finally observed for THF and acetone. This correlates with the visually observed increased scatter when plotting computed versus experimental results (Figure 3f and Supporting Information). Here, the standard deviation of the intercept increases to 1.7 (acetone) and 2.1 (THF) units and its variance to even higher values of 2.9 (acetone) and 4.2 (THF). The uncertainty of the slope on the other hand still remains surprisingly small (σ(slope) ≈ 0.1; σ^2^(slope) ≈ 0.01). This clearly indicates that even in aprotic media the intercept, which strongly depends on the choice of a reliable reference compound, is the main source of errors, whereas the uncertainties related to the computations and relative measurements of the p*K*_a_, which affect the slope, are almost negligible. An interesting outlier is finally DCE. Here, we find two internally completely consistent scaling relations (Figure 3f), which according to the standard deviation and variance of the intercept and slope are rather reliable; e.g., they are significantly lower (orange fit in Figure 3e) and comparable (green fit in Figure 3e) to those obtained for the protic solvents methanol or formamide. However, the existence of these very different fits clearly indicates that the experimental data are unreliable. Similar problems may also be present for other seemingly reliable fits.

Following the above analysis, it is clear that the experimentally reported differences between the solvents^20^ mostly depend on the scatter in the intercept which is heavily affected by the significant uncertainties associated with the choice and reliability of the reference for converting relative to absolute p*K*_a_ values. Taking for example picric acid, the reported p*K*_a_ values vary between −2 for DMSO and 13.7 in DCE.^29^ All other solvents are in this interval with significant differences between them. Similar trends are also observed in the experiments for all other compounds. Using on the other hand the water scaling relations, we are unable to reproduce these trends; e.g., a p*K*_a_ of roughly 8 is found for all aprotic solvents with the exception being THF where it increases to approximately 11. These qualitative trends are not affected by varying the slope of the scaling relationship to 0.75 as approximately found for aprotic media. Instead, they can almost solely be attributed to differences in the intercept of the scaling relation. Using on the other hand the exact individual scaling relationships, the experimentally observed differences also appear in the computed data (see Supporting Information). From a fundamental perspective, the experimentally observed differences could be argued to be expected since all considered solvents display very different properties in terms of hydrogen bonding and polarizability.^125^ However, at the same time it must also be cautioned that the combined influence of these parameters can render very different solvents to behave similarly. This can for example be seen for the proton solvation energy, which is almost identical for all considered aprotic solvents with the exception of THF (see Supporting Information) and thus mirrors the trends in the p*K*_a_ values. Here, the ability for hydrogen bonding compensates for the lower polarizability of the solvent and vice versa. Furthermore, these values do not need to be scaled and are accordingly at least qualitatively comparable. Having said this, it can equally well be argued that the p*K*_a_ values should mirror the behavior of the proton solvation energies and thus must be similar. In this case, the experimental differences would be an artifact of the uncertainties related to anchoring the relative p*K*_a_ values to an absolute scale. Gaining an unbiased computational picture on the trends between solvents would require the use of more complex explicit solvation models in combination with molecular dynamics simulations to determine the correct solvation geometries. In the light of the very similar intercepts observed for the aprotic solvents and assuming additionally that the intercept obtained for water is also approximately applicable for aprotic solvents, eq 18 might be used to approximate the p*K*_a_ value in aprotic media for the SMD solvation model as implemented into Gaussian16 if insufficient (reliable) experimental data are available.
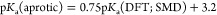
18Naturally, the scaling relationship will change for other computational setups, but a qualitatively equivalent approach can also be used under these circumstances.

Using eq 18 to scale the p*K*_a_ values has at a first glance a detrimental effect as it results in increased deviations between predictions and experiments. This is, however, not a result of the slope of the scaling relation which is rather similar to those observed for the other solvation models but purely due to the differences in the intercept. Indeed, using the exact intercept obtained from the individual scaling relations, the MAEs drop to levels comparable to those observed for the exact scaling relations (see Supporting Information). However, owing to the significant uncertainties associated with the intercepts due to the problems associated with converting the measured relative p*K*_a_ values to an absolute scale, this does not necessarily indicate a poor performance of the general fit (eq 18).

## 4 Conclusions

In summary, we have introduced a novel procedure to predict p*K*_a_ values in nonaqueous solvents which only requires the readily available experimental p*K*_a_ of a reference compound in water. Our method is completely general and can be used with any computational setup and all types of solvation models to predict acid dissociation constants of all compounds in virtually any solvent. The only practical limitation is the availability of suitable implicit solvents or the computational cost of explicit models. The proton solvation energies obtained using this new procedure are robust with respect to the quantum-chemical method and solvation model. Applying this procedure to predict p*K*_a_ values using an implicit solvation model still requires, equivalent to water,^52−55^ empirical scaling relations to map the computed p*K*_a_ values onto the experimental values. This well-known shortcoming could be avoided by the use of more advanced solvation models which would remove the dependence on experimental data in the solvent of interest. Owing to the significant uncertainties in the experimental data, we were unable to perform a detailed benchmark. However, using the scaling relations individually obtained for each considered solvent, the deviations between experiment and computations drop to a level comparable to those observed in water, which highlights the efficiency of our method.

## Supporting Information Available

Summary of deprotonation sequence in water, comparison of unscaled computed and experimental p*K*_a_ in nonaqueous solvents (PDF)
Summary of all compounds including their IUPAC names, scaling relations for correcting the implicit solvation models and the errors associated with them, all computed p*K*_a_’s in all considered solvents, the used solvatochromic parameters, proton solvation energies in water and nonaqueous solvents, relative stability of all considered tautomers, total Gibbs Free energies of all compounds, results of the cross validation (XLS)
Final structures in *xyz* format (ZIP)
