# Supplementary material for: How to Predict the pKa of Any Compound in Any Solvent
Source: ACS Omega. 2022 May 9;7(20):17369–83. doi: 10.1021/acsomega.2c01393 (PMC9134414; doi:10.1021/acsomega.2c01393)
Supplement: Supplementary file 1 — ao2c01393_si_001.pdf [file ao2c01393_si_001.pdf]

Supporting Information: How to Predict the pKa of any Compound in any Solvent

Michael Busch,[*,†] Ernst Ahlberg,[‡] Elisabet Ahlberg,[§] and Kari Laasonen[†]

†*Department of Chemistry and Material Science, School of Chemical Engineering, Aalto University, Kemistintie 1, 02150 Espoo, Finland*

‡*Universal Prediction AB, Gothenburg, Sweden*

¶*Department of Pharmaceutical Biosciences, Uppsala University, Uppsala, Sweden*

§*Department of Chemistry and Molecular Biology, University of Gothenburg, Kemigården 4, 41296 Gothenburg, Sweden*

E-mail: michael.busch@aalto.fi

1 Deprotonation Sequence of Molecules in Training and Test Set in Water

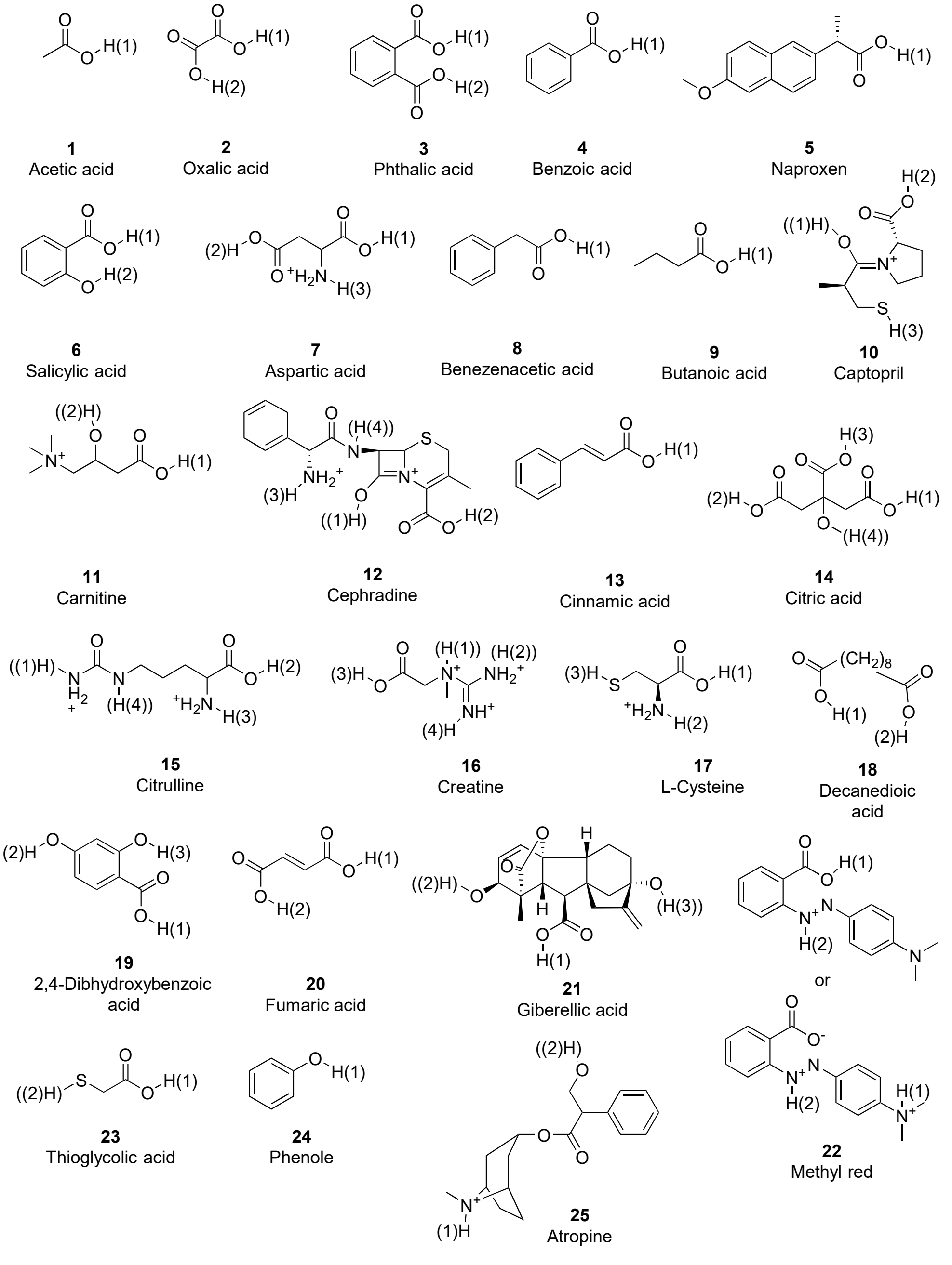

Figure S1: Summary of molecules considered to establish linear free energy scaling relations for accurate pKa computation in water. All considered deprotonation sites are marked by a number stating their sequence (e.g. H(1) is the first proton to leave). Protons marked in brackets (e.g. (H(1))) are not considered in the comparison to the literature values.

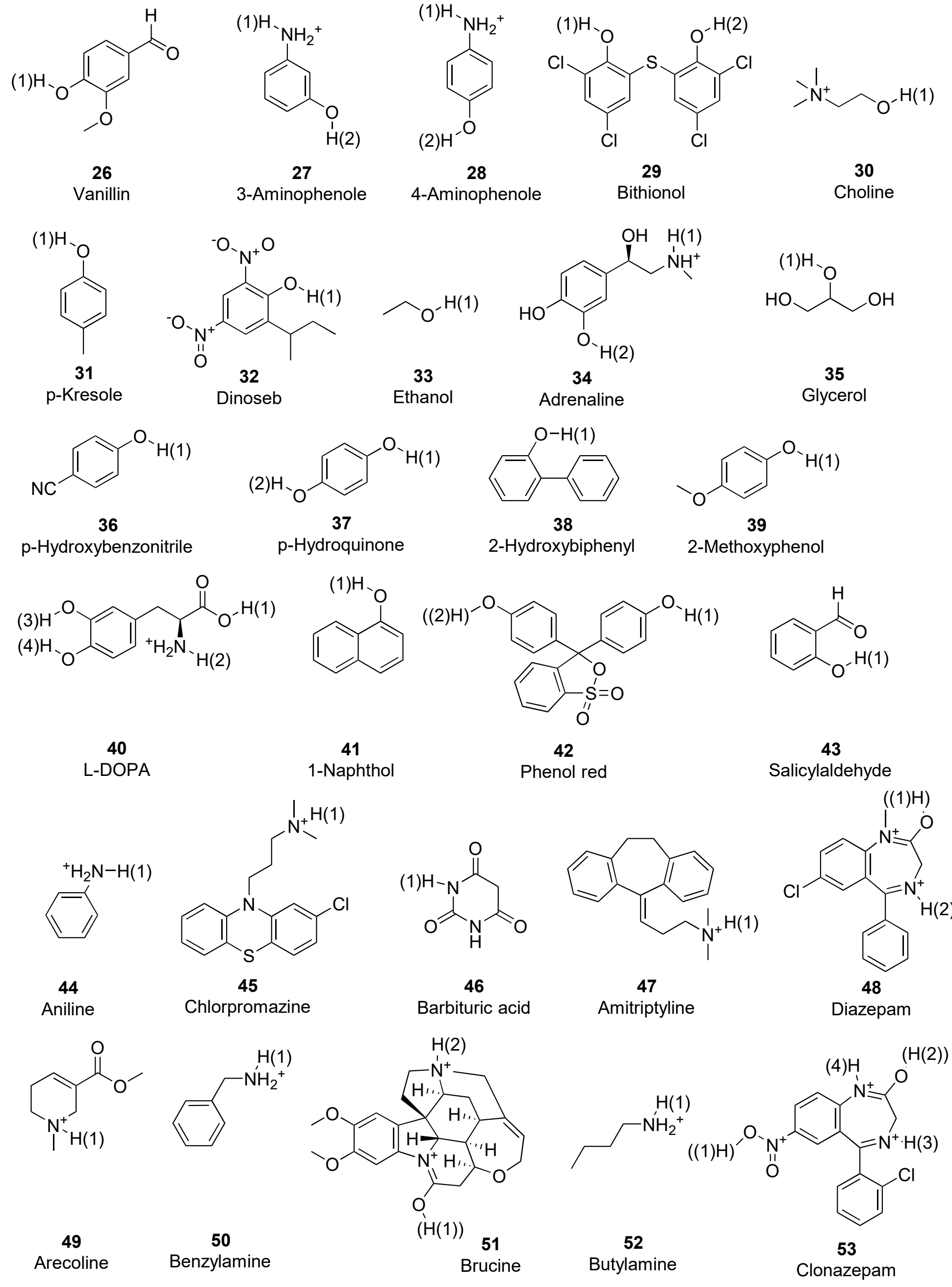

Figure S2: Summary of molecules considered to establish linear free energy scaling relations for accurate pKa computation in water. All considered deprotonation sites are marked by a number stating their sequence (e.g. H(1) is the first proton to leave). Protons marked in brackets (e.g. (H(1))) are not considered in the comparison to the literature values.

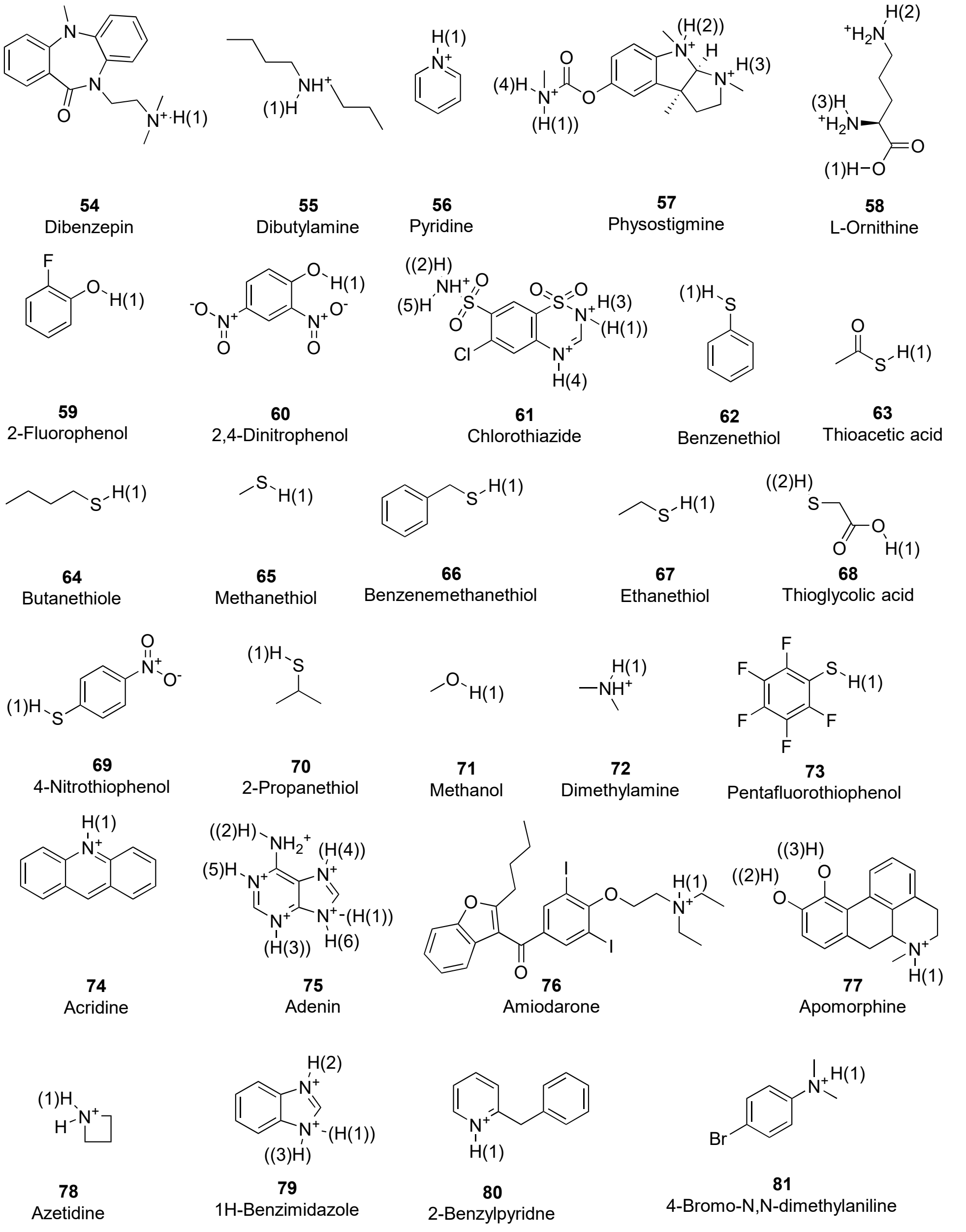

Figure S3: Summary of molecules considered to establish linear free energy scaling relations for accurate pKa computation in water. All considered deprotonation sites are marked by a number stating their sequence (e.g. H(1) is the first proton to leave). Protons marked in brackets (e.g. (H(1))) are not considered in the comparison to the literature values.

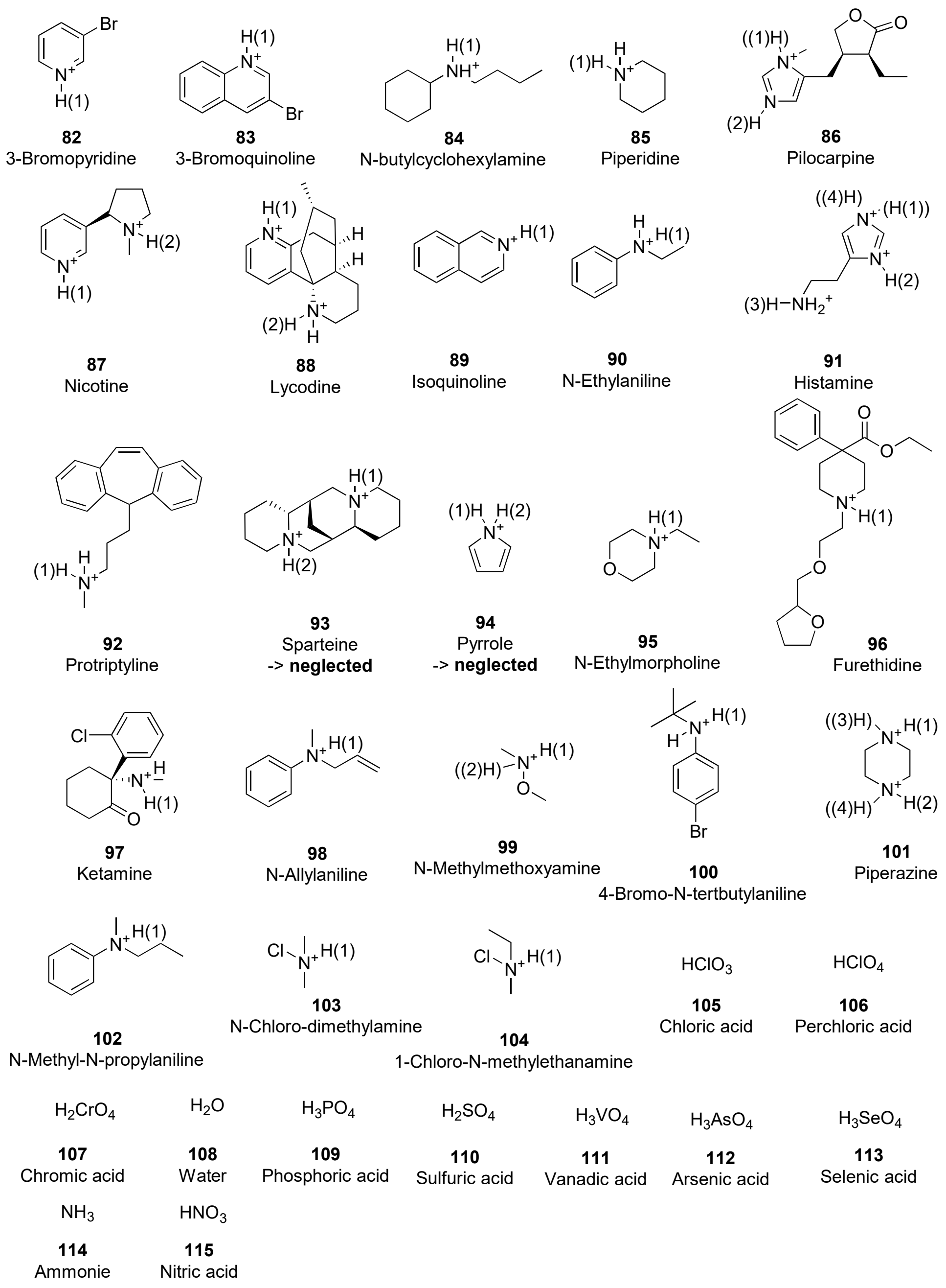

Figure S4: Summary of molecules considered to establish linear free energy scaling relations for accurate pKa computation in water. All considered deprotonation sites are marked by a number stating their sequence (e.g. H(1) is the first proton to leave). Protons marked in brackets (e.g. (H(1))) are not considered in the comparison to the literature values.

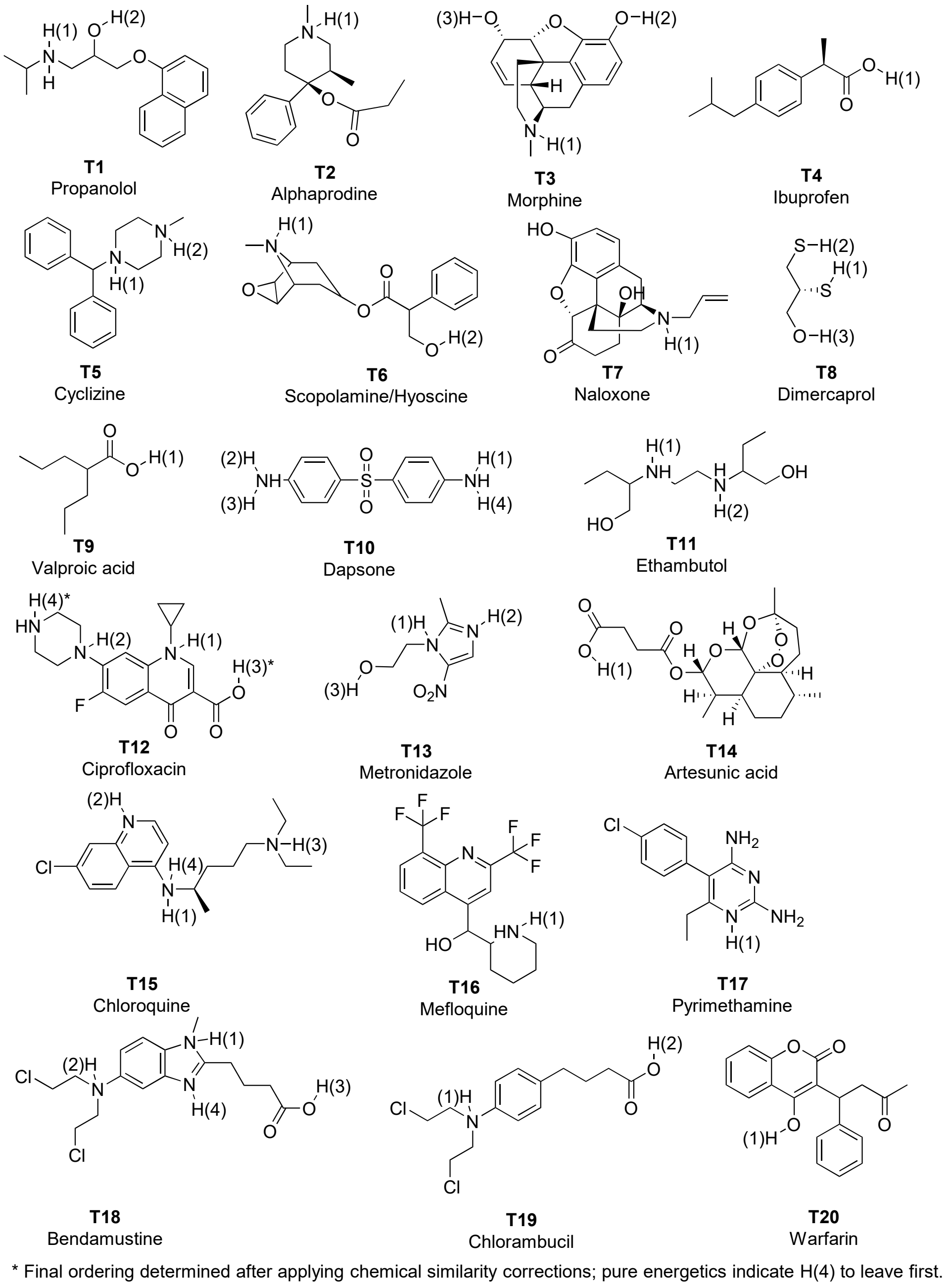

Figure S5: Summary of molecules considered to establish linear free energy scaling relations for accurate pKa computation in water. All considered deprotonation sites are marked by a number stating their sequence (e.g. H(1) is the first proton to leave). Protons marked in brackets (e.g. (H(1))) are not considered in the comparison to the literature values.

2 Unscaled Computed vs. Experimental pKa - Non-Aqueous Solvents

2.1 Isopropanol

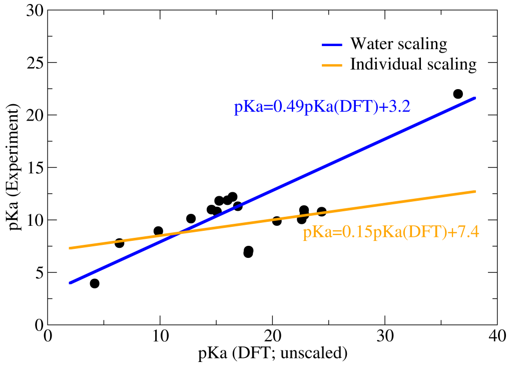

Figure S6: Comparison of unscaled computed and experimental pKa values in isopropanol.

2.2 Acetone

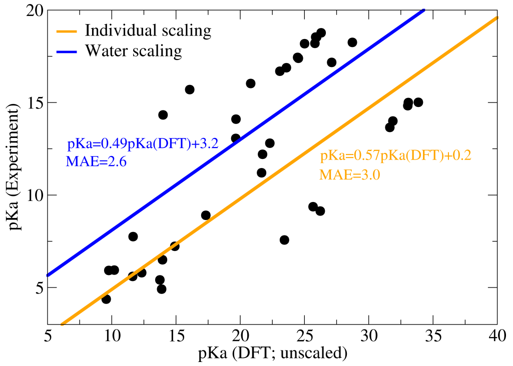

Figure S7: Comparison of unscaled computed and experimental pKa values in acetone.

2.3 Acetonitrile

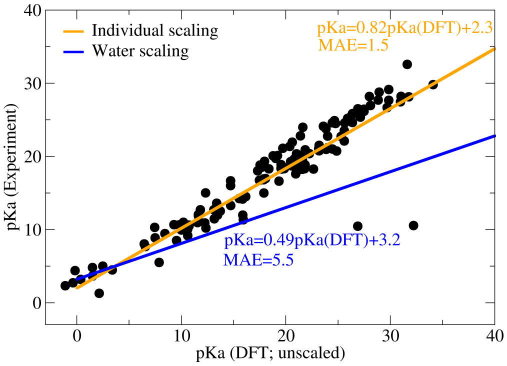

Figure S8: Comparison of unscaled computed and experimental pKa values in acetonitrile.

2.4 1,2-Dichloroethane (DCE)

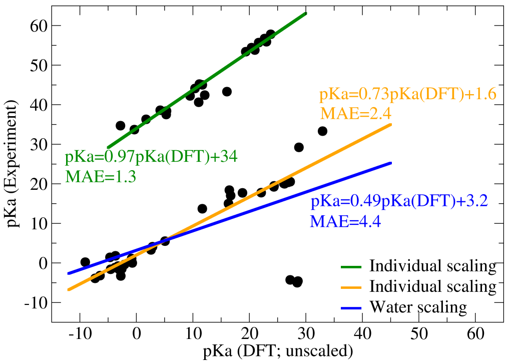

Figure S9: Comparison of unscaled computed and experimental pKa values in 1,2-Dichloroethane (DCE).

2.5 DMF

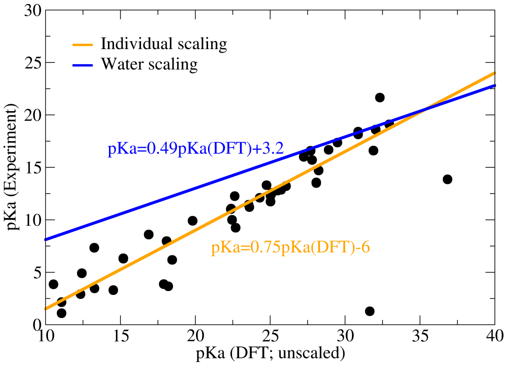

Figure S10: Comparison of unscaled computed and experimental pKa values in DMF.

2.6 DMSO

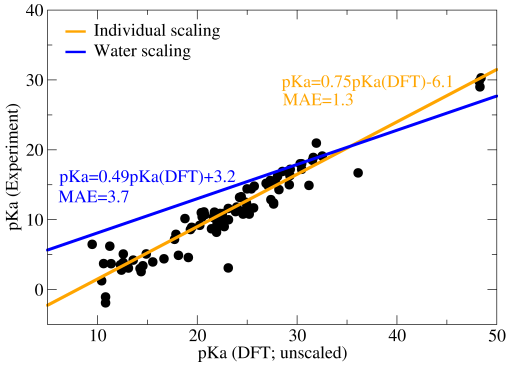

Figure S11: Comparison of unscaled computed and experimental pKa values in DMSO.

2.7 Ethanol

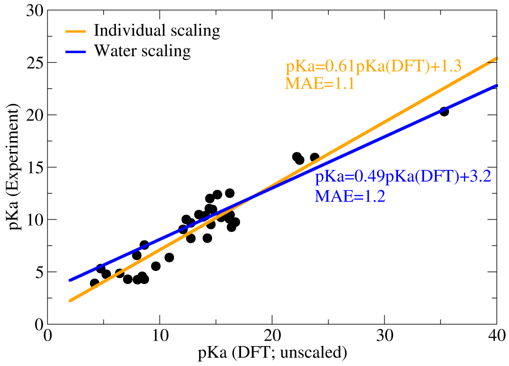

Figure S12: Comparison of unscaled computed and experimental pKa values in ethanol.

2.8 Formamide

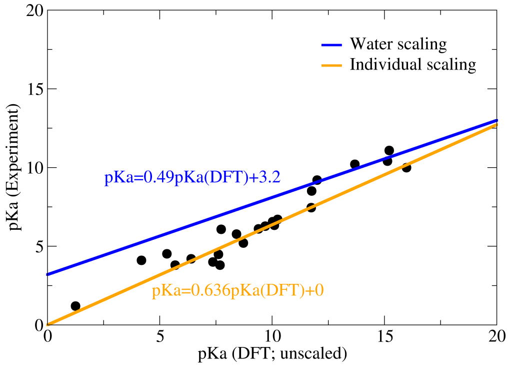

Figure S13: Comparison of unscaled computed and experimental pKa values in formamide.

2.9 Methanol

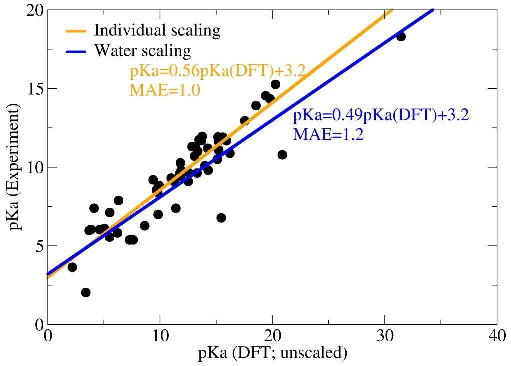

Figure S14: Comparison of unscaled computed and experimental pKa values in methanol.

2.10 Nitromethane

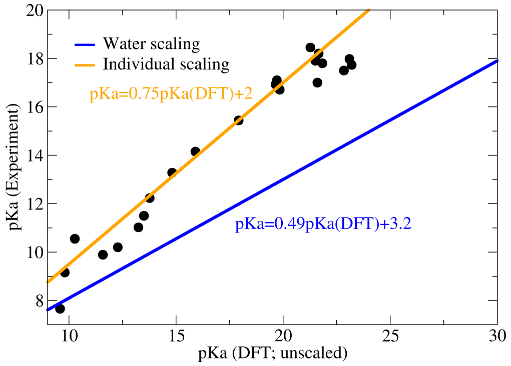

Figure S15: Comparison of unscaled computed and experimental pKa values in nitromethane.

2.11 Pyridine

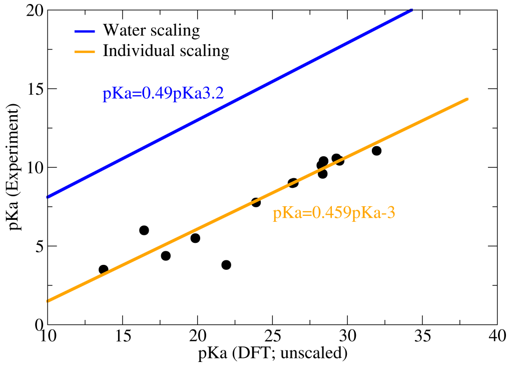

Figure S16: Comparison of unscaled computed and experimental pKa values in pyridine.

2.12 THF

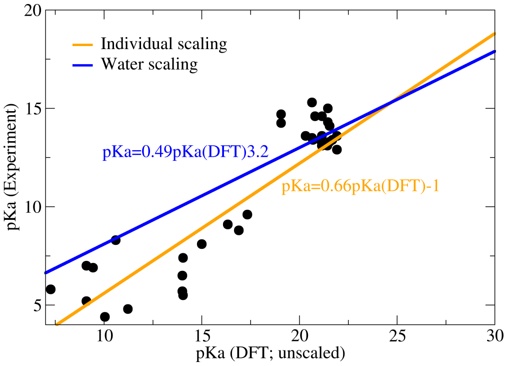

Figure S17: Comparison of unscaled computed and experimental pKa values in THF.
